# Professionals’ Perspectives of Smart Stationary Bikes in Rehabilitation: Qualitative Study

**DOI:** 10.2196/64121

**Published:** 2024-12-31

**Authors:** Julie Soulard, Dahlia Kairy, Roua Walha, Cyril Duclos, Sylvie Nadeau, Claudine Auger

**Affiliations:** 1 Centre de recherche interdisciplinaire en réadaptation du Montréal métropolitain (CRIR) — Institut universitaire sur la réadaptation en déficience physique de Montréal (IURDPM) du Centre intégré universitaire de santé et de services sociaux du Centre-Sud-de-l’Île-de-Montréal (CCSMTL), Université de Montréal, Institut de Réadaptation Gingras Lindsay de Montréal, 6300 avenue de Darlington, Montréal, QC, H3S 2J4, Canada, 1 514-343-6111

**Keywords:** health professionals, attitude, opinion, perception, perspective, cross-sectional, survey, questionnaire, technology, stationary bike, cycling, rehabilitation, physical activity, bike, bicycle, qualitative, content analysis, digital health

## Abstract

**Background:**

Stationary bikes are used in numerous rehabilitation settings, with most offering limited functionalities and types of training. Smart technologies, such as artificial intelligence and robotics, bring new possibilities to achieve rehabilitation goals. However, it is important that these technologies meet the needs of users in order to improve their adoption in current practice.

**Objective:**

This study aimed to collect professionals’ perspectives on the use of smart stationary bikes in rehabilitation.

**Methods:**

Twelve health professionals (age: mean 43.4, SD 10.1 years) completed an online questionnaire and participated in a semistructured interview regarding their needs and expectations before and after a 30-minute session with a smart bike prototype.

**Results:**

A content analysis was performed with inductive coding. Seven main themes emerged: (1) bike functionalities (cycling assistance, asymmetric resistance, and forward and backward cycling), (2) interface between bike and users (simple, user-friendly, personalized, with written reminders during training), (3) feedback to users (user and performance data), (4) training programs (preprogrammed and personalized, and algorithmic programs), (5) user engagement (telerehabilitation, group sessions, music, and automatic suggestion of training), (6) the bike as a physical device (dimensions, comfort, setup, screen, etc), and (7) business model (various pricing strategies, training for professionals, and after-sales service).

**Conclusions:**

This study provides an interpretive understanding of professionals’ perspectives regarding smart stationary bikes and is the first to identify the expectations of health professionals regarding the development of future bikes in rehabilitation.

## Introduction

The World Health Organization estimated that 2.4 billion people are currently living with a health condition and could benefit from rehabilitation [1,2]. Rehabilitation is a key strategy for ensuring healthy lives and promoting well-being for all at all ages [2]. The need for rehabilitation is predicted to increase along with changes in health and population characteristics (ie, longer life expectancy, and prevalence of chronic disease and disability) [2].

New technologies are more than ever used in rehabilitation. There are numerous reasons for developing new technologies for rehabilitation including increasing therapy intensity with less supervision, monitoring the therapy performed by each patient and creating new intervention techniques [3]. For instance, stationary bikes are available in numerous gyms, private clinics, rehabilitation centers and even at home. They are currently used to improve muscle strength, cardiorespiratory endurance, motor skills, mobility, or reduce pain [4-10]. In addition, when used independently as well as under supervision by individuals requiring training [7,11,12], they can significantly increase daily rehabilitation time, which is crucial for recovery [13,14]. However, the current bikes available in rehabilitation centers or clinics have several limitations: few functionalities, basic interface and design, limited universality, and lack of quantitative data on user performance.

With the growing interest in smart technologies and artificial intelligence (AI) in health care, developing smart bikes could enhance rehabilitation by increasing motivation, simulating real-life contexts, offering varied and stimulating environments, and allowing for remote and personalized treatments [15-20]. Health professionals and patients may consider new health care technologies, such as smart bikes, as useful tools [21]. Developing such technologies should, however, include all stakeholders, including health professionals, to meet their expectations and needs, and further facilitate the implementation of these technologies into practice and optimize clinical uptake [22]. Understanding professionals’ needs at the early stages of the development is thus crucial. Therefore, the objective of this study was to collect health professionals’ perspectives regarding smart stationary bikes in rehabilitation to better understand how they could address current rehabilitation challenges.

## Methods

### Study Design

This study used an exploratory qualitative method research design. It is part of a larger project (*MITACS IT27924*) whose objective was to conduct a user-centered needs assessment to (1) inform the development of a new stationary bike and (2) suggest improvements for an existing stationary bike. The data presented in this paper address objective 1 and correspond to phase 2 of the Framework for Accelerated and Systematic Technology Based Intervention Development and Evaluation Research (FASTER) approach. The FASTER approach allows us to generate rigorous and appropriate evidence, specific to technology-based interventions in disability and rehabilitation [23]. Phase 2 corresponds to progressive usability and feasibility evaluation with iterative evaluation of prototypes.

### Ethical Considerations

The research project was approved by the Ethics Review Board on Rehabilitation and Physical Disability of the Centre Intégré Universitaire de Santé et de Services Sociaux du Centre-Sud-de-l’Île-de-Montréal (# 2023‐1697). All participants provided written informed consent. Data of the participants were anonymized and no compensation was provided to them.

### Participants

A purposive sample of rehabilitation professionals (physical therapists, physical rehabilitation assistant, occupational therapists, kinesiologists, and physicians in physical medicine and rehabilitation) was recruited from the authors’ research, clinical, and academic networks by email. All those contacted agreed to participate. They had varied expertise (neurology, orthopedics, geriatrics, and cardiorespiratory) and worked in different settings and rehabilitation phases (private clinic, rehabilitation center, home rehabilitation, community organization, etc). In addition, participants had to live or work in the laboratory area (maximum of 50 km) and have no condition that would affect their pedaling ability.

### Source and Qualitative Data Collection

The data collection involved a two-step process, before and after the bike trial. Prior to the bike trial, an online questionnaire collected sociodemographic information (ie, age, gender, years of experience [<6 years, 6‐10 years, 11‐15 years, 16‐20 years, and >20 years], clinical practice milieu, and clientele). Then, we gathered the experience and expectations of professionals toward stationary bikes for rehabilitation using 4 open-ended questions developed by an interdisciplinary team (ie, researchers with background in occupational and physical therapy and engineering). A preliminary version of the online questionnaire was tested during a pilot session with a professional to improve and refine it.

Few days after the online questionnaire, participants came to the laboratory to test a smart bike prototype. The smart bike was a semirecumbent bike with multiple functionalities. Participants could test pedaling forward, backward, at fixed cadence (passive or active) or fixed resistance (full or partial pedaling cycle, ie, left or right). While pedaling, the user saw on a large screen the interface showing the controls and feedback on the results (torque and cadence).

After the bike trial, a semistructured interview was conducted by the first author (JS, woman, physiotherapist, PhD, postdoctoral student). The interview guide was constructed based on the reference framework “Human Activity Assistive Technology” proposed by Cook and Polgar [24] regarding assistive technology and the user-centered design approach reported by Dabbs et al [25] to define users needs as well as the criteria the technology must meet. The interview guide covered topics such as professionals’ experiences with stationary bikes, relevance, social influence, acceptability, adoption, enabling conditions, financing methods, and safety. At the end of the interview, participants had to choose the 3 most important statements about the bike among a list of statements inspired from the Quebec User Evaluation of Satisfaction With Assistive Technology [26]. The statements included dimensions, comfort, adjustments, efficiency, safety, robustness, aesthetics, as well as items specific for the bike such as pedaling characteristics, ease of use, and the interface between bike and the users. The online questionnaire and interview guide were tested during a pilot session with a professional to improve and refine it. The final versions are presented in Multimedia Appendix 1. The interviews (about 1 hour each) were recorded and transcribed along with the interviewer’s notes.

### Analyses

Qualitative data were analyzed using structured content analysis [27] and categorized into relevant themes emerging from the data. To ensure coding calibration, the main author and a research assistant independently and inductively identified relevant themes emerging from transcripts and interviewers’ notes. Then, the main categories and subcategories were gathered, discussed, and approved. After preliminary coding of three interview transcripts, a final, consensus based, coding guide was developed by part of the research team (JS, DK, SN, and CA). Any differences in opinion regarding the codes were resolved through discussion among team members involved in the data analysis process [28]. The frequency of occurrences was categorized as “most” if identified by at least 7 professionals, “some” by at least 3 professionals, and “few” by less than 3 professionals. The credibility of the analysis was ensured through discussions and interactions with the research team throughout the process (preparation, collection, and analysis).

## Results

### Overview

Twelve professionals were included (7 women and 5 men; age: mean 43.4, SD 10.1 years; Table 1). Most were physical therapists (n=6) and kinesiologists (n=3). They worked in several clinical settings (rehabilitation unit: 7/12, 58%; private practice: n=4, 33%; specialized unit: n=1, 8%) and had varied expertise (neurology: 8/12, 66%; musculoskeletal: 5/12, 42%; geriatrics: 2/12, 17%; cardiorespiratory: 1/12, 8%).

**Table 1.** Rehabilitation professionals’ characteristics (by profession alphabetical order).

Profession	Experience (years)	Area of expertise	Workplace	Rehabilitation phase	Bike use in rehabilitation
Kinesiologist	16‐20	Neurology	Public rehabilitation center; academic	Intensive functional rehabilitation; social and professional reintegration	Sometimes
Kinesiologist	>20	Neurology	Public rehabilitation center; academic	Social and professional reintegration	Often
Kinesiologist	6‐10	Neurology	Private clinic and at home	Prevention	Never
Physician in physical medicine and rehabilitation	<6	Musculoskeletalneurology	Public rehabilitation center	Prevention, acute care and early rehabilitation, andintensive functional rehabilitation	Sometimes
Physical therapist	11‐15	Neurologygeriatrics	Private clinic and at home	Prevention and intensive functional rehabilitation	Often
Physical therapist	>20	Neurology	Public rehabilitation center	Intensive functional rehabilitation	Infrequently
Physical therapist	>20	Cardiorespiratory	Cardiologic public specialized hospital	Prevention, and acute care and early rehabilitation	Often
Physical therapist	16‐20	Musculoskeletal	Private clinic	Prevention, and acute care and early rehabilitation	Never
Physical therapist	<6	Neurology	Public rehabilitation center	Intensive functional rehabilitation	Often
Physical therapist	>20	Geriatricsmusculoskeletal	Geriatric public rehabilitation	Prevention, acute care and early rehabilitation, and specialized care	Infrequently
Physical rehabilitation assistant	>20	Musculoskeletalneurology	Public rehabilitation center	Intensive functional rehabilitation	Infrequently
Occupational therapist	16‐20	Musculoskeletal	Private clinic	Acute care and early rehabilitation	Often

### Professionals’ Perspectives

#### Overview

**Figure 1.**
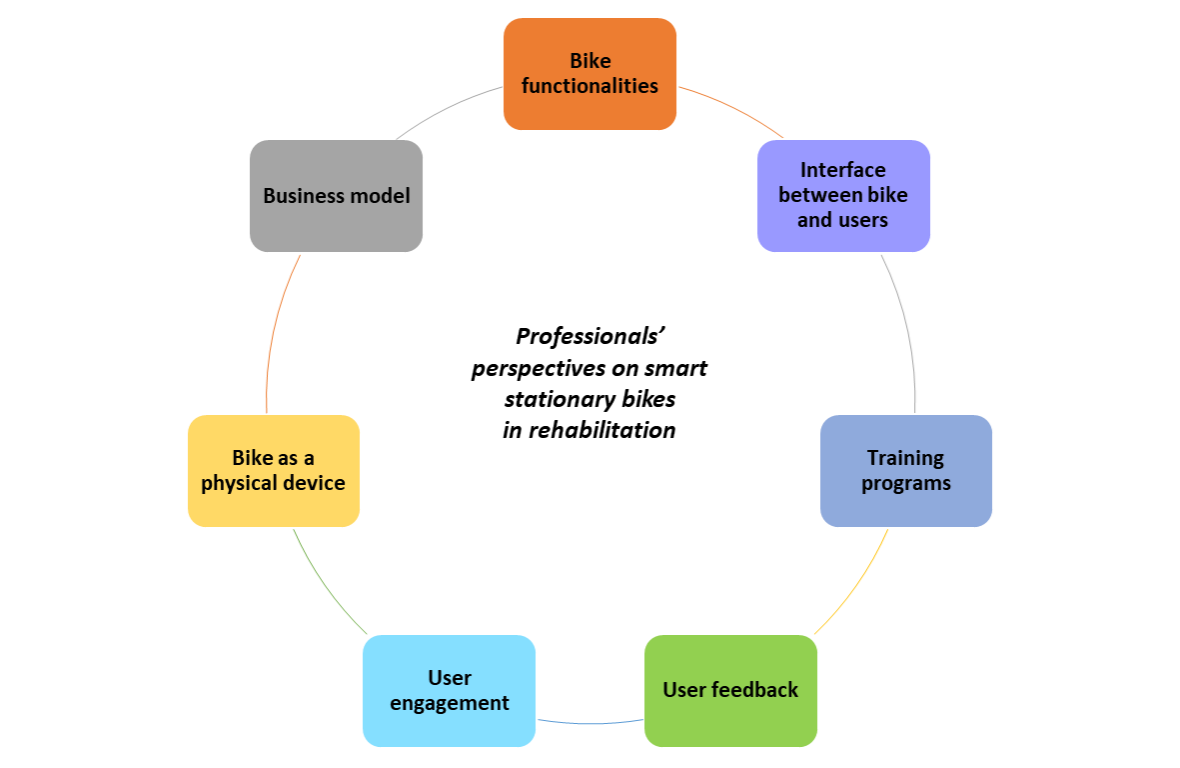
Professionals’ perspectives of smart stationary bikes in rehabilitation.

From the online questionnaire and the semistructured interviews, seven main themes emerged (Figure 1): (1) bike functionalities, (2) interface between bike and users, (3) training programs, (4) user feedback, (5) bike as a physical device, (6) user engagement, and (7) business model.

#### Bike Functionalities

The first theme was related to the bike functionalities. Most of the participants emphasized the importance of controlling pedaling resistance and assistance. Resistance and assistance were most often mentioned, and some professionals mentioned that resistance and assistance should be adjustable during the course of a session “to keep the patient motivated.” These same parameters should also be adjustable independently for each pedal (some). One participant suggested automatic adjustments based on the effort made by the user. Some participants suggested having the option of asymmetrical pedaling, pedaling forward and backward, and one wished to have the possibility of pedaling at very fast speeds (80‐90 revolutions per minute) or to encourage a uniform cycling movement to “improve the work of the lower limb rear muscle chain.” A “realistic mode” was also suggested so that “the pedaling resistance could be representative of outdoor conditions (wind, slope, etc).” Pedaling on this bike could bring sensations, that is, stimulate senses (some), could allow to adjust speed with the possibility of “going around obstacles, accelerating, decelerating quickly,” and to work on trunk balance and coordination while on the bike (n=1). The possibility of using the bike for upper limb pedaling was noted by one professional.

#### The Interface Between the Bike and Users

Most professionals discussed their expectations about the characteristics of the smart bike’s interface. From the outset, some participants emphasized the need for a simple and easy-to-use interface to maximize the independence of users, with a choice of interface languages, simple labeling of the pedaling modes, as well as including infographics (icons, diagrams, colors, and graphs). Most of them also identified the importance of having distinct interfaces for the professional and the client or patient. For professionals, it should be a simple and user-friendly interface (most), including the control of the different modes with one or two primary adjustments on the main page (some), and other possibilities of further adjustments in another page (some). Some professionals suggested that the client or patient interface should offer a “pleasant environment (moving on diverse beautiful roads, even at the seaside),” as well as playful (games with progression) and motivating elements integrated via virtual reality (some). The use of pictograms for user identification was seen as important, especially for patients with cognitive impairment (n=1). It should allow for personalized audiovisual settings to the user’s preference (some). The use of games, pretty pictures, variations and choices in the visuals also seemed important (choice of environment: mountain, city, or country, creation of adapted Tour de France stages; some). Additionally, some professionals identified that it was important to be able to integrate written reminders during training (ie, breathe, relax the shoulders, continue, etc) that were configurable by the professional. A remote control for people who have difficulty reaching the screen was also suggested by one professional.

#### Training Programs

All participants felt that the bike should have the ability to design customized programs to better suit different user profiles. In terms of program content, programs should allow for control of resistance, duration, and pedaling speed. They should contain different modes such as warm-up, workout (at stable or various intensities, with long or short intervals, simulating a slope or a side) and cool down. A “preferred” program (chosen by the client or the professional) could be saved or configured for a “quick start.”

The programs could be adapted to the user’s goals and the results of the initial assessment (some) and be adapted automatically by the smart bike according to the user’s progress using an algorithm (some). To ensure the safety and efficiency of the smart bike, the programs should adapt to the user’s vital signs or perceived effort (n=1). In addition, professionals should be able to view data and change program settings remotely (n=1). These programs should be easily accessible from one session to another (n=2) and allow for comparison between different sessions to quickly visualize the user’s progress (most).

#### User Feedback

Participants repeatedly mentioned the need for multiple feedback elements in order to give information on the user’s and performance’ data while pedaling (most). Participants thought that the user’s data should include aerobic capacity, heart rate, blood pressure, saturation, and different self-report scales such as perceived exertion, pleasure, pain, or dyspnea. Performance data should include speed, duration, power, motor engagement time (ie, in assisted mode, time where the motor had to maintain the targeted pedaling speed), endurance, distance, and energy expenditure. Additionally, where relevant, this data should be available separately for each pedal. For both user and performance data, the idea of having the ability to create graphs and charts for feedback to the patients and professionals was mentioned. This data could be used to track performance during training and between sessions (some) to further improve clinical decision-making. Professionals and users should be able to receive performance reports via email or phone (some) and quantified data should be available for professionals and researchers (some).

#### User Engagement

To increase user engagement on the use of the bike, most professionals suggested: (1) the use of telerehabilitation with the ability for professionals to adjust the smart bike remotely and to monitor symptoms (heart rate and electrocardiogram); (2) the use of music (at the same rhythm as the pedaling); (3) the use of a gamified interface; and (4) sending feedback on performance (strength, time of use, history, and goal achievement) of the day, week, or month and encouragement. Some professionals suggested: (1) the use of group sessions with a resistance adapted to each person, a common pedaling cadence, and a visual allowing the whole group to be seen on the track; (2) automatic suggestion of training sessions adapted to the user and variations in training modes; (3) the connection of the smart bike with the phone (Apple card play or Bluetooth, or plug in the phone) to control the smart bike (facilitate interaction with the interface) and to customize content to each client; and (4) setting up challenges between health care facilities (ie, objective of x km/m).

#### The Bike as a Physical Device

Among the needs and expectations for a smart bike for rehabilitation, several responses concerned the device itself. First, most professionals would like to have the possibility of making various physical adjustments. It was mentioned that “all heights/lengths/angles should be adjustable.” In addition, the adjustments should allow comfort, universality, and safety.

For the screen, it appeared important for professionals to have a large screen and to be able to change its position, orientation, brightness, and font size.

Regarding the pedals, most professionals mentioned that the feet should be maintained with adjustable straps and removing the feet from the pedal should be doable with one hand. One professional identified the need to fix the angle of the pedals to facilitate the positioning of the foot.

The seat should be able to rotate to facilitate transfers from a wheelchair (n=2), or have armrests to assist transfers, and should be adjustable with one hand with a lever that can be placed “on either side for hemiparetic patients.” Also, the use of a wide seat with a comfortable backrest, an adjustable backrest-seat angle, and an adjustable seat height to change the leg positioning was noted by some.

Of these considerations, ease of use and safe patient setup were concerns shared by most participants. Two participants suggested the possibility of having only the crank and pedals, removing the seat to allow people to use the bike from a wheelchair, therefore avoiding a transfer.

As well, various technical devices support should be proposed to adapt to each person, while keeping the possibility of one-handed installation. One professional mentioned that a support for the walking aid (ie, cane) should be added.

Some participants would like a device that is portable in size, for home care or to move it from room to room “for use with hospitalized clients.” It was also mentioned that it should be compact (n=2) and built to ensure it is “durable,” solid (most) and silent (some). The aesthetics of the smart bike was also mentioned by some professionals.

#### Business Model

Finally, according to some professionals, it is important to ensure that the device and its accessories are affordable. Few professionals noted the importance of having a business model with two pricing strategies: one for use at home and one for use in rehabilitation settings. Several business models could be proposed (ie, prepurchased one month rental loan, purchase, short-term [post-op or intensive training] or long-term rental, or rental with option to buy) in order to make it more accessible. The availability of training for professionals was also mentioned by some professionals. Finally, the presence of an after-sales service providing maintenance and technical assistance after purchase was also noted as important (some).

### Important Statement (Quebec User Evaluation of Satisfaction With Assistive Technology)

Lastly, among 10 characteristics presented to them, professionals reported that ease of use (7/12, 58%), interface between bike and users (6/12, 50%), dimensions (5/12, 42%), and pedaling characteristics (5/12, 42%) were the most important categories that should be considered. Safety (4/12, 33%), comfort (4/12, 33%), efficacy (3/12, 25%), and adjustments (3/12, 25%) were less likely to be considered important by the professionals.

## Discussion

### Principal Results

The objective of this study was to explore rehabilitation professionals’ expectations and needs regarding smart stationary bikes in rehabilitation. The qualitative data collected during this study reinforced the importance of considering the stakeholders in designing new technology devices. Most of the devices are designed with a technological focus, with the hope that the device will solve a meaningful clinical issue [29]. However, regarding the close proximity, privileged position, and immersion they have in patient care, professionals are in a unique position to help inform the development of new technologies [30]. Early implication of professionals could further help save time and costs, as modifications can be addressed earlier in the development process [30]. The results of our study highlight the main elements that professionals considered important for the development of a stationary smart bike for rehabilitation and will help developers of future bikes to better meet professionals’ expectations and patients’ needs.

In particular, professionals addressed the smart bike functionalities. While they noted the importance of having the possibility of pedaling against resistance such as all stationary bikes already available in rehabilitation, their expectations were related to innovative modes such as assistance during pedaling or asymmetrical resistance. Indeed, bike training cannot be always be offered during rehabilitation (ie, those unable to pedal against resistance) and if some experimental bikes offer assistance during pedaling [31-34] these experimental bikes are not available widely on the market and the extent to which they respond to clinical needs is unknown. Among the innovative modes identified by professionals, the importance of having an asymmetrical resistance during cycling was noted. Some pathologies lead to asymmetrical involvement of the lower limbs, which will have an impact for instance during walking (ie, orthopedic, neurology, or bariatric populations [35-40]). Given their functional limitations, these populations could benefit from asymmetrical pedaling training. Another main point raised by professionals was the possibility of being able to pedal forward and backward. In clinical rehabilitation settings, very few bikes permit backward pedaling against resistance. However, cycling backward could elicit the work of other muscles [41,42] and further increase functional capacities and cardiorespiratory solicitation [43] in addition to breaking the monotony of cycling forward. Surprisingly, only one professional evoked the possibility of using functional electrical stimulation during cycling. In research, these devices have shown possible applications in rehabilitation [6,44]. It may be possible that most professionals were unaware of these devices; considering they are mainly used in research.

In addition, the bike as a physical device should take into account different characteristics such as comfort, dimensions, safety, and universality. As many professionals of the sample worked with patients in the neurological or geriatric field, practical characteristics were identified regarding setting up the patient (swivel bench or using the bike while sitting on the wheelchair) or adjustments of the bike (adjusting the seat and pedals with one hand). The services related to the bike should also be adapted for home, clinic, and rehabilitation uses (ie, price and financing methods) and available to users (ie, accessibility, users training, technical assistance, and after-sale service) to ensure access for everyone.

Furthermore, being able to monitor users and follow their performance data along with the ability to provide personalized programs were identified as important by most of the professionals. In rehabilitation, the quantification of outcomes is crucial to evaluate the efficacy and efficiency of rehabilitation protocols [45]. In addition, given the importance of rehabilitation intensity and the fact that most individuals in the community receive an insufficient amount of rehabilitation [46], quantifying the time spent in rehabilitation appears important for patients, professionals, and decision makers. Data from the initial assessment, as well as the longitudinal evolution could provide evidence to inform policy and practice, and benefit to the population health [47]. The collected data could even be integrated into electronic health records to improve rehabilitation care [47]. The use of large, comprehensive, and well-maintained data registries (including for instance impairments, activity limitations, and intervention parameters and their evolution) could help in designing tailored rehabilitation programs. Precision medicine aims at delivering “the right intervention, at the right time, in the right setting, for the right person, ultimately bolstering the value of the care that we provide” [48]. Previous studies identified that the efficiency of rehabilitation programs could be improved by using personalized [49,50] or precision rehabilitation [48] including AI. Stationary bikes could provide these types of programs by using quantitative data obtained from the initial evaluation, as well as throughout the rehabilitation process to inform and customize these programs. Even though most professionals addressed the importance of being able to adjust bike parameters manually, none of them alluded specifically to AI. If some barriers in AI use (patients heterogeneity or missing information that could affect the findings) were raised by clinicians in a previous review [51], we believe that AI algorithms including supervised learning should support decision-making and promote patient engagement in the next stages of smart bikes development.

In line with previous studies on technology usability in rehabilitation [52-56], the professionals identified the following prioritizing features: ease of use [52-56], interface between bike and users [53,56], dimensions [52,56], and device characteristics (ie, perceived usefulness) [54-56]. In the user-centered design approach, these characteristics are included in the usability factors, such as learnability, effectiveness, efficiency, errors, flexibility, or user satisfaction [25]. However, our study goes beyond those generic characteristics, by allowing us to delve in the more specific needs related to smart bike development.

### Study Limitations and Perspectives on Future Research

One limitation of this study is that it only considered the professionals’ perspectives. We chose rehabilitation providers for this stage of this study, as they have rich and contextual information regarding patients’ health and functioning during the rehabilitation process [47]. However, given the iterative nature used here for developing the stationary bike, users were involved in different stages of project, including for example in testing the developed bike and rating their satisfaction.

Moreover, there was a short delay between the smart bike trial and the interview which may have limited participant’s reflection. However, at the end of the interview, they were asked to contact us if something new came to mind after their experience. Other limitations were related to the recruited professionals (small sample and part of the researchers’ professional network). To address these limitations, our sample included professionals with a range of characteristics in terms of professional location, professional experience, areas of expertise, and experiences toward cycling in rehabilitation. Moreover, we ensured that the interviews were conducted by a researcher who did not know the participants. Furthermore, the use of semistructured interviews allowed for in-depth data to be collected and better understanding of the person’s needs [57], but it is resource intensive (ie, compared to surveys). Further research is warranted with larger sample sizes, professionals with experience with other patient populations, and different types of smart bikes.

Regarding the development of stationary bikes, the next steps will be to complete phase 2 of the FASTER approach [23] by collecting user perspectives on usability and feasibility of stationary bike prototypes. To this end, qualitative, quantitative, or mixed approaches could be used. Then, the phase 3 of the FASTER approach [23] will consist of intervention deployment and evaluation with users in real-world contexts.

### Conclusions

This study explored the professionals’ perspectives on smart stationary bikes in rehabilitation. Themes related to the bike and its functionalities were identified and allowed us to better capture professionals needs related to bike development. With the arising of new technologies, developers of smart stationary bikes should take these aspects into account to better address unmet health and rehabilitation needs [58].

## Supplementary material

10.2196/64121 Multimedia Appendix 1 French and English versions of the online survey and interview guide.

## Abbreviations

AI: artificial intelligence
FASTER: Framework for Accelerated and Systematic Technology Based Intervention Development and Evaluation Research
