# Supplementary material for: Professionals’ Perspectives of Smart Stationary Bikes in Rehabilitation: Qualitative Study
Source: JMIR Rehabil Assist Technol. 2024 Dec 31;11:e64121. doi: 10.2196/64121 (PMC11705751; doi:10.2196/64121)
Supplement: Multimedia Appendix 1 [file rehab-v11-e64121-s001.docx]

**Supplementary file**

**French version of the online survey and interview guide**

**Questionnaire en ligne**

| 1. Quelle est votre profession ? | □ Physiothérapeute  □ Kinésiologue  □ Ergothérapeute  □ Physiatre  □ Autre. Précisez : _____________________ |
| --- | --- |
| 2. Selon vos fonctions, vous considérez vous principalement : | □ Chercheur ou chercheur-clinicien (recherche>50% du temps)  □ Clinicien  □ Universitaire (enseignement >50% du temps)  □ Etudiant  □ Autre. Précisez : _____________________ |
| 3. A quelle phase de soins et services ci-dessous êtes-vous principalement associé(e) ? | □ Prévention  □ Soins aigus et réadaptation précoce  □ Réadaptation fonctionnelle intensive  □ Réintégration scolaire, sociale et professionnelle  □ Autre. Précisez : _____________________ |
| 4. Dans quel type d’organisation exercez-vous principalement vos fonctions ? | □ Etablissements de santé et services sociaux, précisez : ______________________________  □ Organisme privé  □ Centre de recherche  □ Etablissements d’enseignement |
| 5. Dans quel(s) domaine(s) si situe principalement votre pratique ? | □ Musculosquelettique  □ Neurologique  □ Cardio-respiratoire  □ Pédiatrie  □ Gériatrie  □ Autre. Précisez : _____________________ |
| 6. Dans ce domaine, quelles pathologies rééduquez-vous principalement ? | _______________________________________  _______________________________________ |
| 7. Depuis combien d’années œuvrez-vous comme clinicien ? | □ 0 année  □ moins de 6 années  □ 6 – 10 années  □ 11 – 15 années  □ 16 – 20 années  □ Plus de 20 années |
| 8. Exercez-vous à temps plein ou partiel ? | □ Temps plein  □ Temps partiel, précisez : _________ % |

9. Utilisez-vous le vélo comme moyen d'entraînement avec vos patients ? Si oui, de quelle manière l’utilisez-vous (durée, fréquence, modalités)? Si non, pourquoi ?

10. Etes-vous familiers avec l’utilisation de technologies telles que les dynamomètres (type biodex, cybex) ?

1. Si vous imaginez un vélo créé sur mesure pour vos besoins aujourd'hui, qu'imaginez-vous que ce vélo puisse faire ?
   1. Où, dans quels milieux ?
   2. Quels modes de pédalage aimeriez-vous avoir et pour quels objectifs thérapeutiques ?
   3. Quels ajustements des modes souhaiteriez-vous avoir sur le vélo ?
   4. Quels seraient selon vous les points importants dans la réalisation d’un nouveau vélo
2. Dans le domaine de la santé, l’intelligence artificielle est un outil pouvant aider à la décision clinique par le recueil de multiples données. Comment imaginez-vous l'utilisation d’une composante « intelligente » sur le vélo ? Quel serait son rôle en réadaptation/clinique ?

**Entretiens semi-dirigés**

1. Connaissez-vous d'autres outils similaires au vélo disponibles pour les clients ?

2. Quelle serait la valeur ajoutée de celui-ci selon vous ?

3. S'il y avait des modifications à apporter à cette version du vélo, quelles seraient-elles ?

4. En ajout aux caractéristiques actuelles du vélo, quels sont les éléments qui pourraient motive/encourager le patient à utiliser le vélo ?

5. Sans tenir compte du coût du vélo, est-ce que votre milieu clinique/vous aurai(en) un intérêt à se procurer un tel vélo ? Pour quelles raisons ?

6. Que faudrait-il améliorer et/ou mettre en place pour que le vélo soit accepté/utilisé dans votre milieu de travail ?

7. Un des défis de la recherche est de transférer les connaissances du laboratoire à la clinique. Comment imaginez-vous que ce vélo pourrait être financé ? Combien est-ce que vous pensez que votre milieu ou que le patient accepterait de payer pour ce vélo ?

8. Quels sont pour vous les éléments d’amélioration à prioriser pour une mise sur le marché du vélo dans quelques mois ?

9. Pour terminer, avez-vous d’autres aspects en tête que vous aimeriez aborder sur le vélo ?

10. Vous avez ci-dessous la liste des 10 énoncés de satisfaction auxquels vous venez de répondre. CHOISISSEZ les 3 énoncés qui sont les plus importants pour vous. Inscrivez un X dans les **trois cases** qui correspondent à votre choix.

|  | 1. Dimensions |  | 1. Confort |
| --- | --- | --- | --- |
|  | 1. Ajustements |  | 1. Efficacité |
|  | 1. Sécurité |  | 1. Esthétique |
|  | 1. Solidité |  | 1. Caractéristiques spécifiques de pédalage |
|  | 1. Facilité d’utilisation |  | 1. Interface |

**English version of the online survey and interview guide**

**(free traduction)**

**Online survey**

| 1. What is your profession? | □ Physical therapist  □ Kinesiologist  □ Occupational therapist  □ Physician in physical medicine and rehabilitation  □ Other. Specify: _____________________ |
| --- | --- |
| 2. Based on your duties, do you consider yourself primarily: | □ Researcher or researcher-clinician (research>50% of time)  □ Clinician  □ Academic (teaching >50% of the time)  □ Student  □ Other. Specify: _____________________ |
| 3. With which of the following phases of care and services are you primarily associated? | □ Prevention  □ Acute care and early rehabilitation  □ Intensive functional rehabilitation  □ Academic, social and vocational reintegration  □ Other. Specify: _____________________ |
| 4. In what type of organization do you primarily perform your duties? | □ Health and social service facilities, specify: ______________________________  □ Private organization  □ Research center  □ Educational institutions |
| 5. In what area(s) is your practice primarily located? | □ Musculoskeletal  □ Neurological  □ Cardiorespiratory  □ Pediatrics  □ Geriatric  □ Other. Specify: _____________________ |
| 6. In this area, what conditions do you primarily rehabilitate? | _______________________________________  _______________________________________ |
| 7. How many years have you been working as a clinician? | □ 0 years  □ less than 6 years  □ 6 - 10 years  □ 11 - 15 years  □ 16 - 20 years  □ More than 20 years |
| 8. Do you practice full or part time? | □ Full-time  □ Part-time, specify: _________ % |

9. Do you use cycling as a means of training with your patients? If yes, how do you use it (duration, frequency, modalities)? If no, why?

10. Are you familiar with the use of technologies such as dynamometers (biodex, cybex)?

11. If you imagine a bike custom designed for your needs today, what do you imagine that bike would do?

a. Where, in what settings?

b. What pedaling modes would you like to have and for what therapeutic purposes?

c. What mode adjustments would you like to have on the bike?

d. What do you think would be the most important points in making a new bike?

12. In the field of healthcare, artificial intelligence is a tool that can assist in clinical decision making through the collection of multiple data. How do you imagine the use of an "intelligent" component on the bicycle? What would be its role in rehabilitation/clinical?

**Semi-structured interviews**

1. Do you know of any other bike-like tools available to clients?

2. What would be the added value of this one in your opinion?

3. If there were any changes to be made to this version of the bike, what would they be?

4. In addition to the current features of the bike, what are some things that would motivate/encourage the patient to use the bike?

5. Regardless of the cost of the bike, would you/your clinic have an interest in getting one? For what reasons?

6. What would need to be improved and/or put in place for the bike to be accepted/used in your workplace?

7. One of the challenges of research is to transfer knowledge from the lab to the clinic. How do you imagine this bike could be funded? How much do you think your organization or the patient would be willing to pay for this bike?

8. What do you see as the areas of improvement that need to be prioritized for the bike to be marketed in a few months?

9. Finally, do you have other aspects in mind that you would like to address on the bike?

10. Below is a list of the 10 satisfaction statements. CHOOSE the 3 statements that are most important to you. Put an X in the three boxes that correspond to your choice.

|  | 1. Dimensions |  | 1. Comfort |
| --- | --- | --- | --- |
|  | 1. Fit |  | 1. Efficiency |
|  | 1. Safety |  | 1. Aesthetics |
|  | 1. Solidity |  | 1. Specific pedaling characteristics |
|  | 1. Ease of Use |  | 1. Interface |
